# Evaluating the effectiveness, impact and safety of live attenuated and seasonal inactivated influenza vaccination: protocol for the Seasonal Influenza Vaccination Effectiveness II (SIVE II) study

**DOI:** 10.1136/bmjopen-2016-014200

**Published:** 2017-02-28

**Authors:** Colin R Simpson, Nazir I Lone, Kimberley Kavanagh, Chris Robertson, Jim McMenamin, Beatrix von Wissmann, Eleftheria Vasileiou, Chris Butler, Lewis D Ritchie, Rory Gunson, Jürgen Schwarze, Aziz Sheikh

**Affiliations:** 1 Asthma UK Centre for Applied Research, Usher Institute of Population Health Sciences and Informatics, The University of Edinburgh, Edinburgh, UK; 2 Department of Mathematics and Statistics, University of Strathclyde, Glasgow, UK; 3 Health Protection Scotland, Glasgow, UK; 4 Nuffield Department of Primary Care Health Sciences, Oxford University, New Radcliffe House, Radcliffe Observatory Quarter, Oxford, UK; 5 Cardiff University, Institute of Primary Care and Public Health, Cardiff, UK; 6 Centre of Academic Primary Care, University of Aberdeen, UK; 7 West of Scotland Specialist Virology Centre, Glasgow, UK; 8 Child Life & Health and MRC-Centre for Inflammation Research, The University of Edinburgh, Edinburgh, UK

**Keywords:** EPIDEMIOLOGY, INFECTIOUS DISEASES

## Abstract

**Introduction:**

Seasonal (inactivated) influenza vaccination is recommended for all individuals aged 65+ and in individuals under 65 who are at an increased risk of complications of influenza infection, for example, people with asthma*.* Live attenuated influenza vaccine (LAIV) was recommended for children as they are thought to be responsible for much of the transmission of influenza to the populations at risk of serious complications from influenza. A phased roll-out of the LAIV pilot programme began in 2013/2014. There is limited evidence for vaccine effectiveness (VE) in the populations targeted for influenza vaccination. The aim of this study is to examine the safety and effectiveness of the live attenuated seasonal influenza vaccine programme in children and the inactivated seasonal influenza vaccination programme among different age and at-risk groups of people.

**Methods and analysis:**

Test negative and cohort study designs will be used to estimate VE. A primary care database covering 1.25 million people in Scotland for the period 2000/2001 to 2015/2016 will be linked to the Scottish Immunisation Recall Service (SIRS), Health Protection Scotland virology database, admissions to Scottish hospitals and the Scottish death register. Vaccination status (including LAIV uptake) will be determined from the primary care and SIRS database. The primary outcome will be influenza-positive real-time PCR tests carried out in sentinel general practices and other healthcare settings. Secondary outcomes include influenza-like illness and asthma-related general practice consultations, hospitalisations and death. An instrumental variable analysis will be carried out to account for confounding. Self-controlled study designs will be used to estimate the risk of adverse events associated with influenza vaccination.

**Ethics and dissemination:**

We obtained approval from the National Research Ethics Service Committee, West Midlands—Edgbaston. The study findings will be presented at international conferences and published in peer-reviewed journals.

**Trial registration number:**

ISRCTN88072400; Pre-results.

**Strengths and limitations of this study**

- The study population comprises a large, representative sample of the general population.
- We are developing a large national linked database, which contains anonymised individual patient-level data from general practices, hospitals, virology (real-time PCR) investigations and the death register.
- This is an observational study using routinely collected data, and therefore residual confounding may still be present or unaccounted for. We will measure the levels of unknown confounding required to impact on our study results.

## Introduction

Globally, it is estimated that seasonal influenza is responsible for five million cases of severe illness and 500 000 deaths per year, with an estimated cost to the USA of $87 billion per annum.^1^ ^2^ Worldwide, there are an estimated 90 million new cases of influenza and 1 million cases of influenza-associated severe acute lower respiratory infection among children.^3^ National influenza vaccination programmes delivered by primary care in the community are important for reducing influenza-related illness, hence the considerable investment in this approach. These programmes previously targeted only older people (65+ years) and people considered at risk with chronic diseases, for example, asthma who are particularly susceptible to becoming seriously ill when they get influenza.

Children are important in the transmission of influenza to the populations at risk of serious complications from influenza and diminished circulation of virus has been predicted to improve herd immunity.^4^ Using evidence generated from epidemiological modelling,^5^ and following advice from the Joint Committee for Vaccination and Immunisation^6^ from September 2013, the seasonal influenza vaccination programme has been extended. In addition to the seasonal trivalent and quadrivalent inactivated influenza vaccine for at-risk individuals and those aged 65+ years, the live attenuated influenza vaccine (LAIV) is offered to all children aged 2–17 years (except those severely immunodeficient or those with severe asthma, prescribed immunosuppressive therapy or oral steroids) by primary care clinicians in general practice and schools in Scotland.

Clinical trials have found benefits of LAIV in healthy children under 7 years of age (most for under 3 years old).^7^ ^8^ Efforts to estimate seasonal inactivated influenza vaccine effectiveness (VE) have been largely confined to younger, healthy adults (with few clinical trials showing efficacy in 65+ years and those with chronic disease).^9–11^ Recent observational studies have attempted to estimate the VE in preventing influenza-related illness in general practice patients.^12^ Further studies have examined VE with hospitalisation or death, however they have suffered from bias when using non-specific outcomes,^7^ or have been underpowered when using more specific end points such as laboratory-confirmed influenza, in particular for subgroups being targeted for vaccination (eg, 65+ years and people with at-risk disease such as asthma and pregnant women),^13^ cohort studies (with nested case–control studies) or data linkage-derived estimates of VE have been undertaken with measures taken to overcome many of the confounding issues that otherwise have limited estimations of effectiveness.^14–16^ There is also a need to add to the growing body of evidence with regard to the safety of these vaccines.^17^ Given the ongoing controversy regarding VE and in particular in relation to at-risk groups such as those with asthma,^7^ and the interim recommendation by the Advisory Committee on Immunization Practices (ACIP) not to use the LAIV in the USA during the 2016/2017 seasons,^18^ there is further need for information to help evaluate new policies regarding seasonal vaccine strategies.

This research aims to examine the VE and safety of the seasonal influenza vaccines including LAIV and the inactivated influenza vaccine. We will have access to a unique set of linked databases within a trusted research environment (TRE), which will contain individual patient-level data relating to primary healthcare, acute hospital care, school immunisation data, virological real-time PCR (RT-PCR) laboratory tests and mortality. In contrast to previous observational studies, these richer data sources will provide information on a large number of potential confounders and highly specific laboratory outcome measures in a study cohort sampled from the general population. Our assessment of the effectiveness and the public health impact of a new seasonal influenza vaccination programme seeks to clarify whether such a programme leads to societal benefits, therefore advancing the international evidence base. More specifically, the objectives of this study are to: (1) provide an estimate of the uptake and VE of LAIV administered to children (introduced to the national vaccination programme in 2013); (2) evaluate seasonal influenza VE in at-risk groups (eg, 65+ years, people with asthma, people with other comorbidities and pregnant women); and (3) measure adverse events associated with vaccination.

## Methods

### Study design and population

Vaccine uptake will be reported using serial cross-sectional surveys of each influenza season. The test-negative design (TND)^19^ will be used to measure VE for the RT-PCR outcomes and a cohort study design for non-specific clinical outcomes, for example, hospitalisation or death from influenza or pneumonia.

We will seek to extract data on up to 1.25 million people in Scotland by recruiting 220 general practices into our study. Each patient will contribute person-time to each influenza season while alive and registered with a participating general practice.

### Databases

Data fields extracted from the following databases will be linked deterministically using the Community Health Index (CHI) number—a unique identifier used by the National Health Service (NHS) for the Scottish population. The database linkage and analysis will occur within the National Services Scotland (NSS) TRE by the electronic Database Research and Innovation Service (eDRIS).

*General practice*: Almost all individuals resident in Scotland are registered with a general practice, which provides healthcare services free of charge. Virtually all specialist hospital care services are also free of charge, usually obtained through referral from primary care or, in emergency situations, through patients attending an accident and emergency department. Primary care-based physicians coordinate the influenza vaccination programme for their patients and provide much of the care of patients discharged back into the community by secondary and tertiary care services. Completeness of capture of contacts and accuracy of clinical event coding (using Read codes) has been found to be above 91% among practices in Scotland.^20^ ^21^ The electronic recording of long-term prescribing information by primary care has also been found to be accurate and complete.^22^

*Scottish Immunisation Recall System*: The Scottish Immunisation Recall System (SIRS) is a database that has a record of all children (used nationally from 2002) with scheduled vaccinations. Data on vaccination administration for all children in Scotland are also recorded here.^23^ These data will be used to determine influenza vaccinations that have been administered in schools rather than in primary care.

*Electronic Communication of Surveillance in Scotland*: Data on more than 60 000 RT-PCR tests in total (including an additional 1500 tests per season funded to target 2-year and 3-year olds) have been collated into the Electronic Communication of Surveillance in Scotland (ECOSS) database which is used for the identification of severe disease, outbreaks and long-term trends in the incidence of laboratory-reported infections.^24^

*Scottish Morbidity Record*: The Information Services Division (ISD) maintains a database of all acute hospital discharges and deaths in Scotland, known as the Scottish Morbidity Record 1 (SMR01). All inpatient and day case episodes of care for acute hospitals since 1981 have been recorded in the database. The database is subject to regular validation checks, and the most recent quality assurance report indicated good levels of accuracy (>90%) for the fields used in this study.^25^ Diagnostic information is recorded using the International Classification of Disease V.10 (ICD-10). There are up to six fields the can be used to record diagnoses, with one allocated as the main reason for admission. SMR01 is linked routinely by ISD to the Scottish death register using patient characteristics in a probabilistic matching algorithm with a high degree of accuracy.^26^ Details from death certificates issued for all deaths in Scotland are recorded in the death register, maintained by National Records Scotland (NRS).^27^ Cause of death has been routinely coded using ICD-10 since 2000.

### Study period

**Figure 1:**
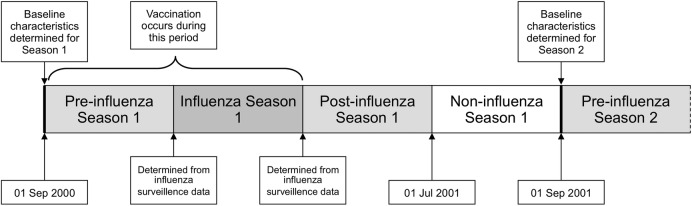
Relationship of first influenza season (2000–2001) to preinfluenza, postinfluenza and non-influenza season periods. Baseline characteristics for each patient are determined on 1 September each year. The earliest date of influenza vaccination varied for each influenza season, but always occurred after 1 September.

Data from 1 September 2000 to 31 August 2016 will be used. This will allow analysis of 16 influenza seasons (2000/2001 to 2015/2016). Each patient will contribute person-time to each influenza season while alive and registered with a participating general practice. For the non-pandemic seasons, each year (1 September to 31 August) will be divided in to four periods (figure 1). The influenza season will be defined for each year using national influenza surveillance data.^28^ The other periods will include a preinfluenza season (starting 1 September), a postinfluenza period, which will end 31 May each year and a ‘non-influenza’ period from 1 June to 31 August (figure 1). In the prepandemic year 2008/2009, there will be no non-influenza period in the summer. The introduction of the influenza vaccination programme for children was phased over successive seasons. Therefore, in season 2013/2014 we will analyse data for children aged 2 and 3 years and those attending primary schools (where pilots were taking place) across Scotland; in seasons 2014/2015 and 2015/2016, we will analyse data for all preschool children aged 2–4 years and all primary school age children (ie, all children aged 2–11).

### Exposure definition

For people in at-risk groups, influenza vaccinations (Trivalent Influenza Vaccine (TIV) and LAIV for preschool children aged ≥2 years) are free and administered by general practice.^29^ Data on influenza vaccination carried out in general practice (including CHI and date of administration) are recorded to enable reimbursement. Information on individuals receiving LAIV in schools is collated in the SIRS database and will be extracted for this analysis.^21^ Vaccination will be used to define exposure status if it is given at a time point between 1 September and the end of the influenza season (figure 1). An individual will be defined as vaccinated 14 days after the seasonal influenza vaccine has been administered.^30^ The time period from the first day of the influenza season to day 14 postvaccination will be defined as ‘unexposed’ and the period from day 14 postvaccination until the end of the influenza season will be defined as ‘exposed’. Therefore, those vaccinated between the start of the preinfluenza period up until 14 days before the influenza season will be defined as ‘exposed’ for the duration of the influenza season. We will exclude participants vaccinated <14 days prior to the RT-PCR test as a sensitivity analysis.

### Patient characteristics and confounding factors

Key characteristics of patients present in each season of the cohort will be included as confounders in the analyses. Confounders will be defined in each year on the first day of the preinfluenza season (1 September).

*Demographics*: Sex, age band and socioeconomic status will be included in all analyses; socioeconomic status will be measured using quintiles of the Scottish Index of Multiple Deprivation (SIMD). SIMD is an area-based measure of deprivation derived from seven domains including income, employment and education.^31^ Rurality in terms of urban/rural location (one large urban, six remote rural) will also be included in the analysis.

*At-risk groups*: At-risk patients are those with certain comorbidities for whom seasonal influenza vaccination is indicated. Patients will be defined as high risk according to national guidance^29^ if they have one or more of the following conditions:

- Asthma;
- Chronic heart disease;
- Chronic kidney disease (including renal transplantation) stages 1–2 and 3–5;
- Chronic liver disease;
- Chronic neurological diseases;
- Chronic respiratory diseases;
- Conditions or drugs causing impaired immune function;
- Diabetes;
- Pregnancy (from 2009/2010);
- Asplenia or dysfunction of the spleen (from 2014/2015);
- Body mass index (for 2015/2016).

*Chronic diseases*: We will include this for our non-specific clinical outcomes and adverse events. Comorbidity will be defined by the 17 disease categories that constitute the Charlson Comorbidity Index.^32^ This Index has been validated in a number of different databases using codes from healthcare databases.^33^ Furthermore, a study has mapped Read codes from a UK general practice database to the relevant Charlson comorbid disease groups, resulting in a model that performed well in the prediction of 5-year mortality.^34^ These codes will be used to identify comorbidities that are present in a patent's record prior to the start of each preinfluenza season (1 September).

*Smoking status*: This will be derived from primary care data (current smoker, ex-smoker, non-smoker) and determined on 1 September each year.

*Previous vaccinations*: A variable will be included for patients who have received seasonal influenza vaccination in previous seasons to account for the possibility of persisting or reduced VE in the subsequent year.^35^ ^36^ Adjustment for previous pneumococcal vaccination at any time in the primary care record prior to 1 September each year will also be undertaken.

*Previous healthcare usage*: Measures of previous healthcare resource use will also capture other aspects of chronic health status and include previous years general practitioner (GP) consultations, prescriptions (repeat) and number of admissions to hospital.

*Functional status*: There is no direct measure of functional status made in any of these national databases. However, individuals who are resident in some form of institutional care setting can be identified from the primary care database. This will also be used as an indicator of more severe functional limitation.

*Practice*: Accounting for the clustering of patients within GP practices, we will investigate whether practice characteristics (eg, training practice, list size, type of contract, etc) are associated with vaccine uptake.

### Studies

#### RT-PCR study

We will target the recruitment of primary care practices involved in the Health Protection Scotland (HPS) Pandemic Influenza Primary Care Reporting (PIPER) sentinel-swabbing scheme, whereby practices are encouraged to obtain nasal/throat swabs from patients of all ages who have symptoms suggestive of influenza. Each general practice is requested to submit five swab samples per week (seven from season 2015/2016) to the West of Scotland Specialist Virology Centre (WoSSVC), Glasgow Royal Infirmary, Glasgow, UK for RT-PCR testing for a range of respiratory pathogens on any patient presenting for consultation in the practice with influenza symptoms across all ages independent of whether the patient has or has not been vaccinated. Data are also collated by HPS on RT-PCR testing patients from swabbing carried out in primary and secondary care for routine diagnostic purposes outside the sentinel scheme. All RT-PCR data on positive and negative tests are held by HPS in the national laboratory database—ECOSS database. WoSSVC is a WHO accredited National Influenza Center, which participates in the quality assurance programme to maintain this status. From 1999, the RT-PCR testing used to confirm respiratory virus type has been found to be highly sensitive for influenza A (H3, H1) and B diagnosis.^37^ Improvements to RT-PCR since 2003 include the development of multiplex testing increasing the number of pathogens tested per PCR assay, however the high sensitivity of these tests remain unchanged.^38^

#### Non-specific clinical outcomes study

To determine the effect of vaccination status on influenza-related primary care consultations, hospital admissions and deaths, secondary analyses will be undertaken using non-specific clinical outcomes derived from primary and secondary care. Influenza-like illness (ILI) and otitis media consultation will be derived from the general practice database. Hospitalisation and cause of death from influenza or pneumonia will be derived from SMR01. We will also include in our secondary analysis a range of asthma-related outcomes, for example, asthma-related consultations/symptoms or measurements, hospitalisations/deaths, rescue medications, etc.

#### Unmeasured confounding and instrumental variable analyses

In addition to Simonsen's Framework, we will also assess the robustness of our results by modelling the effect of an unmeasured confounder on our VE estimates in sensitivity analyses, an approach used previously^39^ and being widely adopted to help explain the role of unknown confounding in observational analyses.^40^ We will vary three factors: the prevalence of the confounder in the vaccinated population, its prevalence in the unvaccinated population and the increased risk of the outcome attributable to the confounder.^41^ The use of influenza vaccine coverage by geographical area has been found to be a strong and valid instrumental variable (IV), which can be used to account for confounding.^42^ Rather than compare patients with respect to whether they received influenza vaccination, this IV behaves like natural randomisation of patients to regional vaccination groups that differ in their likelihood of receiving influenza vaccination. The NSS TRE is an important development in this respect, and we have received permissions to extract granular postcode/geocoding data required to test the validity of this IV analysis in the SIVE II database. We will therefore explore the suitability of vaccination uptake in geographically distinct Health and Social Care Partnership areas or other suitable Health Board areas as a suitable IV. To be valid, this IV needs to be related to exposure status (ie, vaccination status) and not have an independent effect on outcome other than by ways mediated through the exposure.^43^ Furthermore, the IV should not be related to any variables that confound the relationship between exposure and outcome. If an association with confounders is demonstrated, it is assumed that the IV is associated with unmeasured confounders and is therefore not valid. If the IV fulfils these criteria, it can be used in analyses to produce unbiased estimates of VE by accounting for unmeasured confounding.

#### Adverse events associated with vaccination study

We will explore the use of self-controlled study designs to estimate the risk of adverse events following influenza vaccination.^44^ The assumption underlying these designs is that, in the situation where the adverse event is related to vaccination, the occurrence of an adverse event in the period after vaccination is greater than periods in the same patient that are temporally unrelated to vaccination.^45^ These methods have the advantage of controlling for all fixed individual-level confounders as comparisons are within the same individual rather than between vaccinated and unvaccinated populations. The time period at risk for an adverse event (risk interval) and time period not at risk (control interval) will be determined separately for each outcome.^46^

### Statistical analysis

Baseline characteristics will be summarised by vaccination status for the whole cohort using mean, median or proportion where appropriate together with a measure of dispersion. We will evaluate the baseline characteristics of those tested and not tested using RT-PCR. Missing data will be reported for each variable. A 5% significance level will be used for hypothesis tests for the primary outcome. All p values will be two sided. All analyses will be undertaken in R (V.3.2.3; R Core Team (2015). R: A language and environment for statistical computing. R Foundation for Statistical Computing, Vienna, Austria. URL https://www.R-project.org).

#### Annual and pooled analyses

We will initially analyse each of the 16 influenza seasons separately for the primary outcome. However, a pooled analysis will be performed if increased precision is required (particularly for less common outcomes or where analysing subgroups of patients). We will test the heterogeneity of the vaccine effect over the seasons, by testing for interaction between vaccine status and year for the outcomes. If appropriate, we will then pool the data to give a more powerful analysis than the stratified results. Where heterogeneity occurs, however, the pooled analysis can be restricted or abandoned. In this pooled analysis, we will account for the within-person correlation resulting from repeated measures on the same individual in different influenza seasons.

#### Vaccine uptake

Vaccine uptake will be modelled through logistic regression. ORs (adjusted for age, sex, clinical risk group and deprivation) for differences in proportion of vaccine uptake between different groups of patients (eg, sex, age, SIMD categories and at-risk groups) and for investigating trends in vaccine uptake will be calculated. Vaccine uptake and 95% CIs will be calculated. Practice characteristics will be incorporated by using a multilevel approach.

### Vaccine effectiveness

#### RT-PCR outcomes

For VE using information from linked virological RT-PCR swab data (a binary event) we will carry out a nested case test-negative control study.^19^ Influenza positivity will be compared with no influenza among patients who have ILI symptoms and tested for influenza. The primary analysis will be through a logistic generalised additive model where the effects of gender, age, socioeconomic status (SIMD^33^) and being in an at-risk morbidity group are adjusted for (a TND study). A spline function for time during each season will be included to model the background rate of influenza and correct for any potential bias associated with the proportions of test-negative and test-positive patients at different periods. VE will be measured by comparing the results from swabs taken after vaccination among those vaccinated to swabs taken among those unvaccinated at the time the swab is collected. Vaccination will be used to define exposure status if it was given at a time point between the 1 September and the end of the influenza season (figure 1). The adjusted estimate of VE=(1**−**OR)×100, where the OR is derived from the coefficient of vaccine status in the model. In our main analysis, we will assess only the first dose when two doses are given. We will carry out analysis stratified by influenza A (H1—including pandemic influenza and H3 subtype where recorded) and B.

We will carry out a number of sensitivity analyses for the primary end point:

1. *Non-sentinel* versus *sentinel*: We will explore the validity of using laboratory-confirmed influenza tests from non-sentinel primary care and secondary care sources versus sentinel primary care practices. Patient characteristics of individuals swabbed in non-sentinel primary care practices and secondary care will be described and any interaction between source of swab and outcome will be tested.
2. *Negative controls*: As there should be no VE for influenza vaccine for these outcomes, we will explore the use of laboratory-confirmed infections (currently 10 respiratory viruses including rhinovirus and adenovirus) tested using multiplex RT-PCR at the same time as the influenza RT-PCR.
3. *LAIV VE in younger/immunodeficient*: For younger vaccine-naïve or immunodeficient children receiving LAIV, we will account for any second dose in our model.

#### Non-specific clinical outcomes

We will estimate VE for non-specific clinical outcomes: primary care consultations for ILI; all-cause emergency hospitalisation and death; and emergency hospitalisation and death due to influenza/pneumonia. Hospital admissions and consultations can have multiple events and each event will be counted.

For this proposal we have adopted methods we found in previous studies to be optimal for measuring VE and accounting for bias and confounding.^47^ Adjusted rate ratios of VE for prevention of hospitalisation/death/general practice consultation will be derived from time-dependent Cox models, taking into account the time at risk and the possibility of multiple events (not for death). Models will include a cluster term to account for intrapractice correlation. These models will adjust for gender, age, deprivation and clinical risk group and exposure to vaccination in each season included as a time-dependent covariate. Each season, individuals will begin in the unvaccinated group (and will accumulate time at risk) until 14 days after the receipt of the vaccine; then they will switch to the vaccinated group.

In all models used to estimate the VE, we will adjust for variables associated with the receipt of a vaccination and effect modifiers, such as vaccinations, consultations and hospitalisation in the previous influenza season, SIMD, urban/rural status, smoking status, Charlson score and pregnancy.

### Sample size

We expect a final total sample size of up to 1.25 million from 220 practices. Using data from the PIPER 2014/2015 cohort, which has 263 000 individuals, 16% are aged 2–17, and 18% aged 65+.^48^ Vaccine uptake among children aged 3–12 years is 60% and vaccine uptake among those aged 65+ is 70%. Linked to this cohort from all virology tests in Scotland were 1745 RT-PCR tests overall; 331 RT-PCR tests among 2–17 years old; 366 RT-PCR tests among those aged 65+. This gives a multiplier ratio of around 5:1 from the PIPER cohort to the SIVE II cohort and this is used to estimate the numbers of RT-PCR tests expected each year. We expect 1800 RT-PCR tests per year among those aged 65+, 1650 laboratory tests per year among those aged 2–17. We expect 1745/12×5=630 patients with asthma swabbed per year as about 12% of the population is treated for asthma.

Using data generated from the SIVE project,^49^ we estimate a vaccination rate of 60% among children targeted for receipt of LAIV and a swab positivity rate of 20% among the unvaccinated. This should give 90% power to detect a VE of 31% based on 1650 swabs in one season. Pooling data over two seasons give an estimated 3300 swabs in children eligible for vaccination and a 90% power to detect a VE of 22%.

For 65+ year olds targeted for receipt of TIV where there is a vaccination rate of 70% and a swab positivity rate of 10% among the unvaccinated we anticipate an 80% power to detect a VE of 39% where we estimate that there will be 1800 swabs each year in the later years. During the peak influenza activity when swab positivity might increase to 20% there is a 90% power to detect a VE of 31%. About 1 in 12 of the population is treated for asthma^50^ and we anticipate 1260 swabs among patients with asthma in final two seasons. Assuming 40% are vaccinated and that the swab positivity is around 15% gives 80% power for a VE of 35%. For an influenza type/subtype which has 50% of the total cases, then the detectable VEs will be 40–50% with 90% power. For a type/subtype which has about 25% of the cases, then the detectable VEs will increase to over 60% with 90% power. These powers above do not take into account design effects for the clustering of patients within GP practices. Analyses of the historic PIPER cohorts^48^ has revealed design effect of <7% and this serves to increase the detectable VE by about two percentage points.

### Dissemination

The study findings will be presented at international conferences and published in peer-reviewed journals (STROBE and RECORD will be used to guide transparent reporting).
